# Antigen-Loaded Extracellular Vesicles Induce Responsiveness to Anti–PD-1 and Anti–PD-L1 Treatment in a Checkpoint Refractory Melanoma Model

**DOI:** 10.1158/2326-6066.CIR-22-0540

**Published:** 2023-01-10

**Authors:** Rosanne E. Veerman, Gözde Güclüler Akpinar, Annemarijn Offens, Loïc Steiner, Pia Larssen, Andreas Lundqvist, Mikael C.I. Karlsson, Susanne Gabrielsson

**Affiliations:** 1 Division of Immunology and Allergy, Department of Medicine, Karolinska Institutet, Solna, Stockholm, Sweden.; 2 Department of Clinical Immunology and Transfusion Medicine, Karolinska University Hospital, Stockholm, Sweden.; 3 Department of Oncology-Pathology, Karolinska Institutet, Stockholm, Sweden.; 4 Department of Microbiology, Tumor and Cell Biology, Karolinska Institutet, Stockholm, Sweden.

## Abstract

Extracellular vesicles (EV) are important mediators of intercellular communication and are potential candidates for cancer immunotherapy. Immune checkpoint blockade, specifically targeting the programmed cell death protein 1 (PD-1)/programmed death-ligand 1 (PD-L1) axis, mitigates T-cell exhaustion, but is only effective in a subset of patients with cancer. Reasons for therapy resistance include low primary T-cell activation to cancer antigens, poor antigen presentation, and reduced T-cell infiltration into the tumor. Therefore, combination strategies have been extensively explored. Here, we investigated whether EV therapy could induce susceptibility to anti–PD-1 or anti–PD-L1 therapy in a checkpoint-refractory B16 melanoma model. Injection of dendritic cell–derived EVs, but not checkpoint blockade, induced a potent antigen-specific T-cell response and reduced tumor growth in tumor-bearing mice. Combination therapy of EVs and anti–PD-1 or anti–PD-L1 potentiated immune responses to ovalbumin- and α-galactosylceramide–loaded EVs in the therapeutic model. Moreover, combination therapy resulted in increased survival in a prophylactic tumor model. This demonstrates that EVs can induce potent antitumor immune responses in checkpoint refractory cancer and induce anti–PD-1 or anti–PD-L1 responses in a previously nonresponsive tumor model.

## Teaser

In mice with checkpoint-refractory melanoma, antigen-loaded EVs induce antitumor immune responses. Prophylactic immunization with EVs sensitizes tumors to anti–PD-L1 and leads to prolonged survival, indicating potential use of EVs against checkpoint-refractory cancers.

## Introduction

Extracellular vesicles (EV) are lipid bilayer nanoparticles released by cells and play an important role in both cell-to-cell communication and modulation of immune responses. Depending on their cell of origin, EVs can deliver immunostimulatory or immuno-inhibitory messages between cells (1). Furthermore, they can carry multiple cargoes, including tumor-derived antigens, and deliver them to antigen-presenting cells (APC; refs. 2–4). Armed with both immunostimulatory capacities and tumor antigens, EVs from APCs might be ideal to combat intratumoral heterogeneity, a condition often resulting in therapy resistance (5). Moreover, EVs have been tested in several clinical trials, which show that they can promote natural killer (NK) cell function; however, further optimization of EV therapy is needed to better stimulate T cells (4, 6, 7). In previous studies, we observed that EVs from bone marrow–derived dendritic cells (BMDC) loaded with natural killer T (NKT)–activating α-galactosylceramide (αGC), along with ovalbumin (OVA)-induced NK and NKT cell activation, boost antigen-specific T- and B-cell responses and reduce tumor growth (8–10). Moreover, immunization with αGC- and antigen-loaded EVs induces long-term memory, indicating that these EVs can be used in vaccination settings (11).

In the past decade, the use of therapeutic antibodies against immune-negative, co-stimulatory molecules, such as programmed cell death protein 1 (PD-1) and programmed death-ligand 1 (PD-L1), has revolutionized the cancer field. Despite the remarkable success of PD-1/PD-L1 signaling inhibition, only approximately 20% to 30% of patients respond to antibody monotherapy (12). Several mechanisms have been proposed to explain this. It has been shown that anti–PD-1 or anti–PD-L1 treatment is ineffective when administrated to tumors with low immune cell infiltrates, so-called “cold” tumors (13, 14). For anti–PD-1/anti–PD-L1 treatment to be effective, tumor cells need to be recognizable to T cells. Tumors can escape immune killing by downregulating MHC class I and II, which prevents their recognition by T cells (15–18). Furthermore, tumors recruit and promote the differentiation of immunosuppressive cells, such as myeloid-derived suppressor cells, tumor-associated macrophages, and cancer-associated fibroblasts, which create a hostile environment for infiltrating immune cells (19). These immunosuppressive cells express PD-L1 and other immune inhibitory markers and release immunosuppressive cytokines such as IL10 and TGFβ and thereby suppress the infiltrating immune cells, making the checkpoint blockade ineffective (20–22).

To improve or induce responses to anti–PD-1/anti–PD-L1 treatment in a checkpoint-unresponsive cancer, a therapy that promotes both T-cell infiltration into tumors and immunogenicity of the tumor itself is desirable. Therefore, we hypothesized that EVs loaded with αGC and OVA, by means of activating both innate and adaptive immune cells, have the potential to induce the desired antitumor response in nonresponsive cancers. Subsequently, the “EV primed” tumor would be sensitized to anti–PD-1/anti–PD-L1 treatment, resulting in an effective response.

To address this, we used a mouse model of melanoma in which tumor cells expressed the model antigen OVA, and which is insensitive to checkpoint blockade monotherapy (23, 24). Mice were treated therapeutically or prophylactically with OVA and αGC-loaded BMDC-derived EVs and/or anti–PD-1/anti–PD-L1. Treatment with anti–PD-1 or anti–PD-L1 alone had no effect, whereas EV treatment induced upregulation of MHC class I and PD-L1 on tumor cells and enhanced tumor infiltration by total immune cells, as well as antigen-specific T cells, indicating a desired immune response. When EVs were combined with immune checkpoint therapy, we observed a potentiated effect on immune responses, which also resulted in increased survival in a prophylactic model. These data indicate that EVs can promote responsiveness in a tumor that is initially insensitive to anti–PD-1/anti–PD-L1 treatment, making treatment with EVs and anti–PD-1/anti–PD-L1 combination a promising strategy for the clinic.

## Materials and Methods

### Mice

For generation of BMDCs and for tumor experiments, 7- to 9-week-old female C57BL/6nTac mice (Taconic, Tornbjerg, Denmark) were kept under specific pathogen-free conditions at the Karolinska Institutet's animal facility. All experiments were approved by the Stockholm Animal Ethics Committee. An acclimatization period of at least 5 days was applied before experiments were performed.

### BMDC generation

BMDCs were generated from C57BL/6nTac mice as previously described (25). Briefly, bone marrow cells collected from the tibia and femur were cultured in BMDC culture medium consisting of RPMI 1640 media (Hyclone), supplemented with 10% heat-inactivated FCS (Gibco), 2 mmol/L l-glutamine, 1X penicillin–streptomycin, 1 mmol/L sodium pyruvate (all Hyclone), 50 μmol/L β-mercaptoethanol (Sigma), recombinant mouse GM-CSF (10 ng/mL, BioLegend) and IL4 (2 ng/mL, Peprotech) for 6 days. On day 6, OVA (300 μg/mL; Sigma) and αGC (100 ng/mL; KRN-7000; Funakoshi) were added to the culture and incubated overnight. On day 7, the cells were washed, followed by 48-hour culture in the presence of the same media as mentioned above, with the addition of LPS (30 ng/mL, Sigma) and containing EV-depleted FCS (produced by spinning 30% heat-inactivated FCS at 100,000 × *g* for 16 to 18 hours and removing the pelleted bovine EVs) instead of regular FCS. BMDCs were stained on day 9 for surface markers at an antibody concentration of 1 μg/mL at 4°C for 30 minutes and acquired using a FACS Canto II (BD Bioscience). Data were analyzed using FlowJo software (version 10, BD). Antibodies used included: anti-CD11b (clone M1/70), anti-CD11c (clone N418), anti-CD14 (clone Sa14–2), anti-CD9 (clone MZ3), anti-CD63 (clone NVG-2), anti-CD81 (clone Eat-2), anti-CD40 (clone 3/23), anti-CD80 (clone 16–10A1), anti-CD86 (clone GL-1), anti-ICAM-1 (clone 3E2), anti-MHCI (clone AF6–88.5), anti-MHCII (clone M5/114.15.2), anti-CD1d (clone 1B1), anti–PD-L1 (10F.9G2), anti–PD-L2 (clone TY25), anti–PD-1 (clone 29F.1A12), anti-CTLA-4 (clone UC10–4B9), rat IgG2a (RKT2758), rat IgG2b (RTK4530), hamster IgG (HTK888) and mouse Ig2a (MOPC-173), all from BioLegend.

### EV isolation

To enrich for small EVs/exosomes, supernatants from BMDC cultures were collected on day 9 of BMDC culture, after the removal of cells by centrifugation for 10 minutes at 300 × *g*. Supernatants were transferred to new tubes and further centrifuged at 3,000 × *g* for 30 minutes to remove cell debris. Microvesicles/large EVs were removed by centrifugation for 30 minutes at 16,500 × *g*, and supernatants were subsequently filtered through 0.22-μm pore-sized filters (Nordic Biolabs). EVs were isolated by 2-hour ultracentrifugation at 100,000 × *g*. After washing with PBS, the EV pellets were resuspended in a small volume of PBS. The protein concentration was measured using a Micro BCA Protein Assay Kit (Thermo Scientific) according to the manufacturer's instructions, using the Perkin Elmer Enspire 2300 Multilaber reader. EVs were stored at a concentration of 0.5 μg/μL to 5 μg/μL in PBS at −80°C until further use.

### Transmission electron microscopy

EVs were analyzed by transmission electron microscopy (TEM) with a negative ion capture. An aliquot of 3 μL from each sample was added to a grid with a glow-discharged, carbon-coated supporting film for 3 minutes. Excess solution was removed using filter paper. The grid was rinsed with distilled water for 10 seconds and dried with filter paper, followed by staining with uranyl acetate in water for 7 seconds. After air-drying the grid, the samples were examined at 80 kV using a Hitachi HT 7700 EM (Hitachi), and images were obtained using a Veleta camera (Olympus).

### Nanoparticle tracking analysis

The size distribution and particle concentration of EVs were measured using nanoparticle tracking analysis (NTA) (LMH10HSB system, Nanosight, Malvern). Samples were diluted with 30 kDa filtered PBS to reach a 5 × 10^8^ to 5 × 10^9^ particles/mL concentration and were subsequently analyzed in a 60-second recording, with an infusion rate of 50, camera level of 13, and detection threshold of 3. Five independent EV batches were analyzed.

### EV phenotyping using flow cytometry

Streptavidin-coated magnetic particles (SVMS-40–10, Spherotec) were diluted to 0.5 mg/mL and incubated with 10 μg of biotinylated anti-mouse CD9 (clone EM-04, BioSite Flow) overnight at room temperature. After washing with PBS, the beads were blocked with 100 mmol/L glycine for 30 minutes at room temperature, followed by washing with 0.5% BSA (Sigma)/PBS. Anti-CD9–coated beads were incubated with 2 μg EVs per μL of beads overnight at room temperature. EV-coupled beads were stained for surface markers (200 μL of 1 μg/mL antibody) in PBS for 30 minutes at room temperature. Samples were acquired using a FACS Canto II (BD Biosciences) and analyzed using FlowJo software (version 10, BD). Antibodies used: anti-CD63 (clone NVG-2), anti-CD81 (clone Eat-2), anti-CD86 (clone GL-1), anti-ICAM-1 (clone 3E2), anti-MHCI (clone AF6–88.5), anti-MHCII (clone M5/114.15.2), anti-CD1d (clone 1B1), anti–PD-L1 (10F.9G2), anti–PD-1 (clone 29F.1A12), rat IgG2a (RKT2758), rat IgG2b (RTK4530), hamster IgG (HTK888), and mouse Ig2a (MOPC-173), all from BioLegend.

### Western blot

Total protein was extracted from EVs and BMDC cells diluted in 1X RIPA buffer (Bio-Rad), followed by three rounds of 15-second vortexes between five-minute sonication cycles. Protein concentration was determined using the DC protein assay (Bio-Rad) and the Perkin Elmer Enspire 2300 Multilaber reader. EV or cellular proteins (20 μg) were boiled in 1X reducing Laemmli Sample Buffer (Bio-Rad) for 5 minutes at 95°C. Proteins were run on a Mini-Protean TGX precast gel (any kDa; Bio-Rad) and blotted onto Trans-Blot Mini PVDF membranes (Bio-Rad) using the Trans-Blot Turbo Transfer system (Bio-Rad). After blocking for two hours with 5% nonfat milk/PBST at room temperature, membranes were incubated overnight at 4°C with primary antibodies anti-OVA (1: 4,000; clone 0220–1682G, AO Serotec/Bio-Rad), anti-MHCII (1: 4,000; clone ab180779, Abcam), anti-calnexin (1:1,000; ab10286, Abcam), and anti-actin (1:10,000; Sc1616, Santa Cruz), followed by detection with a donkey anti-rabbit (1: 10,000; GE Healthcare). The bands were visualized using enhanced chemiluminescence buffer (GE Healthcare) and analyzed using the ChemiDoc MP Imaging System and Image Lab software version 4.1 (Bio-Rad), respectively.

### BMDC stimulation *in vitro*

BMDCs were cultured as described above, without the addition of OVA or αGC, on day 6. On day 7, the cells were washed and seeded at 150,000 cells/well in a 96-well flat-bottom plate in 200 μL of BMDC culture medium. Next, the cells were cultured for 24 hours in the presence of the following stimuli: 100 ng/mL LPS, 2 μg/mL OVA, 2 μg/mL αGC, and 5 μg/mL or 25 μg/mL BMDC-derived EVs loaded with OVA and αGC. After 24 hours, the cells were harvested, washed, and stained for surface markers with 1 μg/mL antibody, followed by flow cytometry analysis. Antibodies used: anti-CD86 (clone GL-1), anti-ICAM-1 (clone 3E2), anti-MHCI (clone AF6–88.5), anti-MHCII (clone M5/114.15.2), anti-CD1d (clone 1B1), anti–PD-L1 (10F.9G2), all from BioLegend.

### Cell lines

B16/OVA melanoma cells (F1, OVA-secreting) were cultured for five days (two passages) in RPMI 1640 (Hyclone) containing 10% heat-inactivated FCS (Gibco), 2 mmol/L l-glutamine, 1X penicillin–streptomycin, and 400 μg/mL geneticin (all from Hyclone) in an incubator at 37°C and 5% CO_2_. B16/OVA were received from Dr. J. Frelinger, University of Rochester, Rochester, NY. The culture was tested for *Mycoplasma* contamination using the Lookout Mycoplasma PCR Detection Kit (Sigma-Aldrich).

### OVA-secreting B16 melanoma tumor model

Where indicated, B16/OVA melanoma cells were first stimulated with 20 ng/mL recombinant mouse IFNγ (Peprotech) for 24 hours before inoculation. For all tumor models, 100,000 B16/OVA melanoma cells (F1) were injected subcutaneously into the right flank of C57BL/6nTac mice. Tumor development was monitored by measuring with a caliper every 2 to 3 days, and mice were sacrificed depending on the experimental setting, i.e., at day 20 or when the tumor size reached 1,000 mm^3^. Tissue collection was performed after sacrificing the mice. In the sacrifice and therapeutic models, mice were intravenously injected with 100 μL of PBS as a control, 40 μg EV alone or in combination with intraperitoneal injection of 125 μg anti–PD-1 (clone RMP1–14, InVivoMAb) or 225 μg anti–PD-L1 (clone 10F.9G2, InVivoMAb) 5 days after tumor inoculation. Anti–PD-1 and anti–PD-L1 intraperitoneal injections were repeated on days 8 and 12, along with intravenous EV injections on day 12. When mice were sacrificed, as specified, spleens and tumors were collected. B16 tumors were cut in small pieces (∼10 mm^3^) and added to a 6-well plate with each well containing 1 mL RPMI with 0.5 Wünsch U/mL liberase TL (Roche). The plates were kept at 37°C for 30 minutes. Next, spleens and digested tumors were passed through a 100-μm cell strainer (Corning) to create single-cell suspensions. Red blood cells were removed using ACK lysis buffer [made in-house: 1 L MiliQ water containing 8 grams NH_4_Cl (Scharlau), 1 gram KHCO_3_ (Scharlau), and 0.1 mmol/L EDTA (Sigma)]. Subsequently, the tumor cells and splenocytes were washed and resuspended in 5- and 3-mL PBS, respectively. Blood was collected from the inferior vena cava and added in Microtainer collection tubes (Gold SST, BD Bioscience) and centrifuged at 10,000 × *g* for 10 minutes to isolate serum, which was used to analyze OVA-specific antibody responses via ELISA, as described below. In the prophylactic model, mice were immunized before tumor inoculation by injecting intravenously 100 μL of PBS or 40 μg of EVs on days 0 and 14. Fourteen days after the second injection, mice were inoculated with the tumor cells. On days 3, 6, and 9, 125 μg of anti–PD-1 or 225 μg of anti–PD-L1 was intraperitoneally administered to mice, and tumors, spleens and blood were collected, processed as described, and used for immunological assays. The following exclusion criteria were defined *a priori* for specific outcome measures: (i) open ulceration of the tumor (flow cytometry); (ii) sudden death, ulceration of tumor or illness of mouse requiring sacrifice before approaching the experimental endpoint (tumor < 800 mm^3^; tumor growth, survival, and flow cytometry); (iii) no/very small tumor (tumor growth curve in the therapeutic setting and tumor flow cytometry). For mice without tumors, flow cytometry was not performed in the therapeutic setting and is presented separately for the prophylactic setting.

### FACS analysis

FACS analysis was performed using 200-μL tumor or splenocyte single-cell suspensions per antibody panel. Cells were first incubated at 4°C for 30 minutes in PBS with anti-CD16/CD32 Fc block (BD Biosciences) at 1: 100 and live/death staining (Zombie Aqua, BioLegend) at 1: 1,000, or Live/Dead fixable blue dead cell stain (Invitrogen). Next, cells were washed and stained at 4°C for 30 minutes in PBS with antibodies against CD45 (clone 30-F11), B220 (clone RA3–6B2), CD3 (clone 17A2), CD8 (clone 53–6.7), MHC-I (clone AF6–88.5), PD-1 (clone RPM1–30), PD-L1 (clone 10F.9G2), NK1.1 (clone PK136), CD11b (clone M1/70), Ly6G (clone 1A8) and F4/80 (clone BM8), all from BioLegend, at 1 μg/mL. PE-labeled H-2Kb - SIINFEKL pentamer (ProImmune) was used at 2.5 μL per staining. In some sets of experiments, absolute cell numbers were calculated by adding 10 μL counting beads (1 × 10^6^ beads/mL, Spherotech) to each sample before acquisition.

### ELISAs

The surface expression of OVA on EVs was determined using ELISAs. EVs were coated onto ELISA plates (Nunc Maxisorp flat bottom plate, Thermo Scientific) overnight at 4°C at a concentration of 5 μg/mL. For the standard curve, the wells were coated with 0.7 to 500 ng/mL OVA. Mouse anti-OVA (clone 3G2E1D9, LSbio) was added at 1:1,000 v/v ratio and incubated for 2 hours at room temperature, followed by 1 hour at room temperature with secondary antibody (anti-mouse IgG HRP; Southern Biotech) at 1:2,000 v/v ratio. TMB substrate (Mabtech) was used for detection, according to the manufacturer's protocol, and the reaction was stopped by adding 100 μL of 1 mol/L H_2_SO_4_ to each well. The plates were read at 450 nm using a Perkin Elmer Enspire 2300 Multilaber reader.

OVA-specific IgG antibody responses in serum collected from the therapeutic model were determined by coating plates with 10 mg/mL OVA protein overnight at 4°C. Serum was added to wells starting from 1:10 dilution in PBS incubated for 2 hours at room temperature, and anti-OVA IgG was detected by incubating 1 hour with anti-IgG-AP (Southern Biotech) at room temperature, followed by the addition of AP-substrate buffer [in-house: 0.1 mol/L glycine (Sigma), 1 mmol/L MgCl_2_ (Sigma), and 1 mmol/L ZnCl_2_ (Sigma), pH 10.4] containing 0.5 mg/mL phosphatase substrate (Sigma). The absorbance was read at 405 nm using the Enspire 2300 Multilaber reader.

### Elispot

IFNγ secreted by splenocytes (harvested from the therapeutic model) upon *in vitro* restimulation was determined using anti-mouse IFNγ ELISPOT according to the manufacturer's instructions (Mabtech). In brief, PVDF plates (Merck-Millipore) were coated overnight at 4°C with anti-IFNγ at 1:200 in PBS (100 μL/well). After coating, excess capture antibodies were washed away with PBS, and the plates were blocked for 2 hours at 37°C in ELISPOT medium consisting of RPMI 1640 containing 10% heat-inactivated FCS, 2 mmol/L l-glutamine, and 1X penicillin–streptomycin. Next, 200,000 splenocytes per well were plated in ELISPOT medium. The cells were incubated for 19 hours at 37°C with the following stimuli: 2 μg/mL concanavalin A (Sigma), 2 μg/mL CD8 OVA peptide SIINFEKL, 2 μg/mL CD4 peptide OVA323–339 (both from Innovagen), 2 μg/mL whole OVA protein (Sigma), or 2 × 10^5^ OVA-expressing B16 cells. After stimulation, the plates were washed, and biotin-labelled anti-IFNγ (Mabtech) was added at 100 μL/well (1:1,000 in 0.5% FCS containing PBS) for 2 hours at room temperature. Next, the plates were washed, and SA-ALP (Mabtech) was added at 100 μL/well (1:1,000 in 0.5% FCS containing PBS) for 2 hours at room temperature. Finally, the plates were washed and developed by adding 100 μL/well BCIP solution (Mabtech). All ELISPOT plates were read using an AID iSpot FluoroSpot Reader System and analyzed using the AID ELISPOT software (Autoimmun Diagnostika).

### Data availability

The data generated in this study can be provided by the corresponding author upon request.

## Results

### Characterization of BMDC-derived EVs

**Figure 1.**
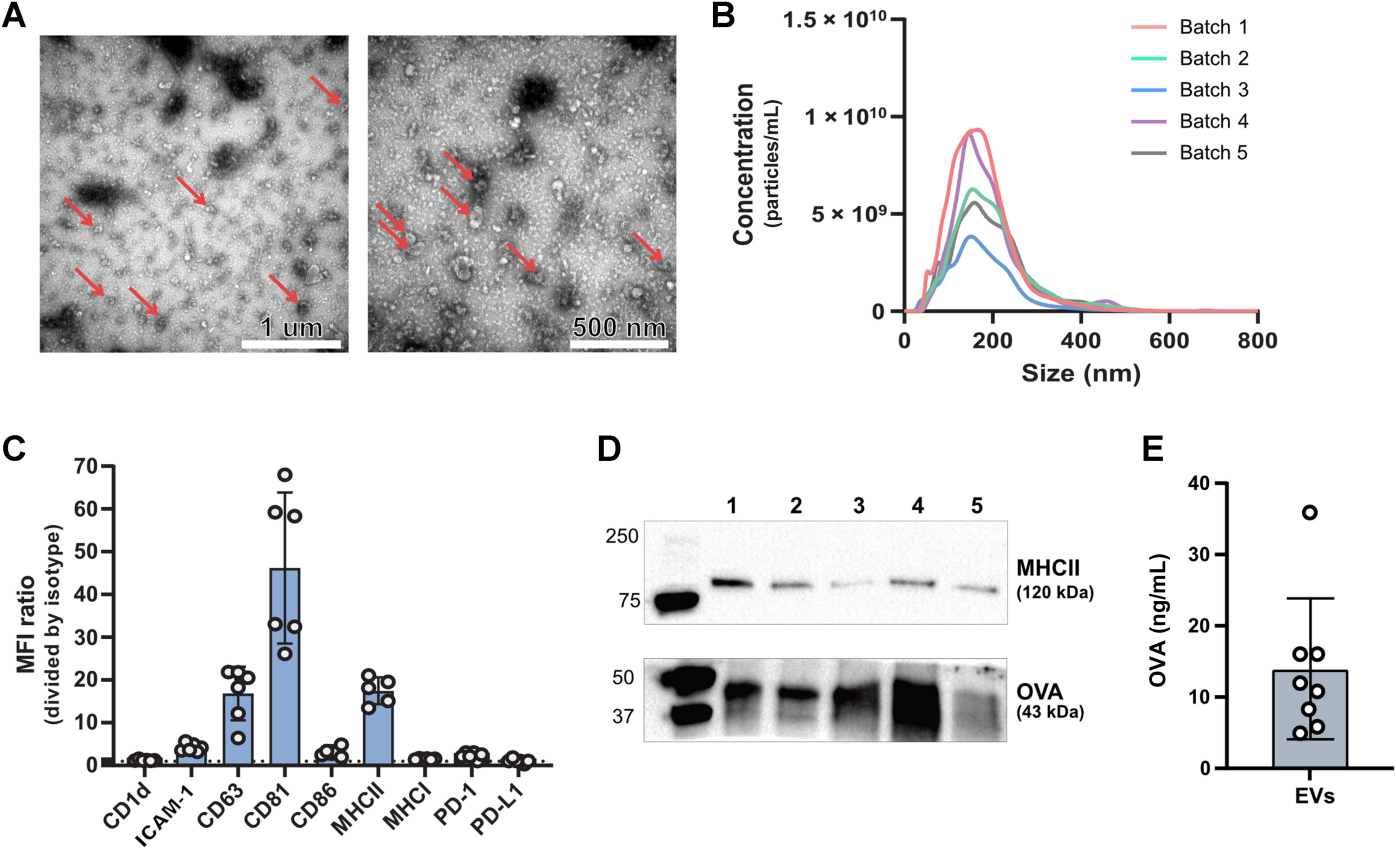
Characterization of BMDC-derived EVs. BMDC-derived EVs were characterized using multiple methods. **A,** Morphologic analysis of EVs was performed by TEM. Two different magnifications are shown. **B,** Five EV batches, shown individually, were subjected to NTA analysis to determine EV size (nm), and a mean and mode size 184 and 157 nm, respectively was measured. **C,** EVs were bound to anti-CD9–coated magnetic beads, stained for surface markers as indicated, and detected by flow cytometry. The data are shown as the mean fluorescence intensity ratio between the specific antibody and its corresponding isotype control. A signal above 1 (dotted line) was considered positive. Data are presented as mean ± SD, *n* = 6. **D,** Western blot analysis was performed on proteins extracted from the five EV batches for the presence of MHC class II and OVA. **E,** The presence of OVA on the surface of EVs was determined by ELISA. Data are shown as the mean ± SEM, *n* = 8.

Mature BMDCs were generated by 48-hour LPS stimulation and subsequently characterized by flow cytometry, which showed the presence of MHC class I and MHC class II, as well as the co-stimulatory molecules CD80, CD86, and ICAM-1 (Supplementary Fig. S1A). EVs were isolated from mature BMDCs and characterized (size and morphology) using TEM according to the Minimal Information for Studies of Extracellular Vesicles Guidelines (26). Vesicle preparations from BMDCs displayed EV characteristics, such as small cup-shaped particles in the range of 80 to 200 nm (Fig. 1A, red arrows). Additional TEM images from a second EV batch are displayed in Supplementary Fig. S1B. The size distribution of the EVs was further analyzed using NTA. NTA results confirmed the presence of EVs with an average size of 184 nm (range 172–195 nm) and a mode size of 157 nm (range 146–170 nm; Fig. 1B). Bead-based flow cytometry of EVs coupled to anti-CD9 coated beads revealed the presence of tetraspanin molecules CD63 and CD81, in addition to ICAM-1, CD86, and MHC Class II molecules on the surface (Fig. 1C). The presence of MHC Class II and OVA antigen on EVs from APCs (26) was further confirmed by Western blot analysis (Fig. 1D). The presence of the OVA antigen was also verified by ELISA (Fig. 1E). Pure vesicle isolation was demonstrated by the absence of the endoplasmic reticulum-associated marker calnexin in EV lysates (Supplementary Fig. S1C).

### EVs combined with checkpoint blockade induce antigen-specific immune responses in a melanoma model

**Figure 2.**
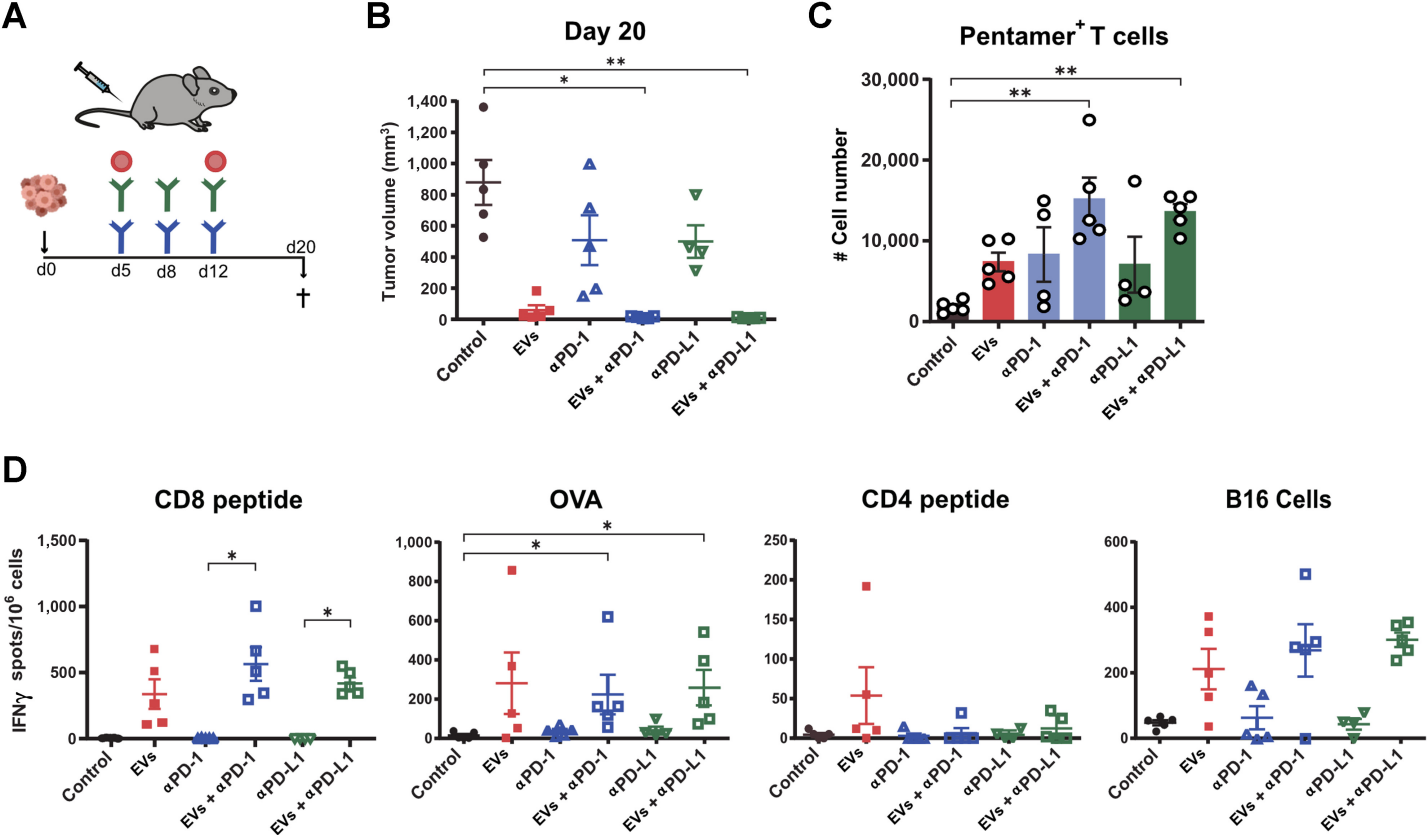
Combination treatment of EVs and anti–PD-1/anti–PD-L1 improves antitumor immunity on day 20 after tumor inoculation. **A,** Mice with OVA-expressing B16 melanoma were treated with PBS or EVs intravenously and anti–PD-1/PD-L1 intraperitoneally as indicated. Mice were sacrificed on day 20 for further analysis. **B,** Tumor volume was measured on day 20. **C,** The total number of OVA-specific CD8^+^ T cells in the spleen was determined using flow cytometry. **D,** ELISPOT assay was performed to detect IFNγ-secreting cells after *in vitro* restimulation with the CD4 peptide OVA_323–339_, CD8 peptide SIINFEKL, whole OVA protein, or B16 melanoma cells. The data represent one experiment. Dots represent a single mouse, 4 to 5 mice per group, and data are presented as the mean ± SEM, except for figure 2C which is displayed as the mean ± SD. Data were analyzed using the Kruskal–Wallis test with Dunn test for multiple comparisons. *, *P* < 0.05; **, *P* < 0.01; and ***, *P* < 0.001.

Our previous studies showed that OVA- and αGC-loaded BMDC-derived EVs delayed OVA-expressing B16 melanoma tumor growth in mice (9, 11, 27). To test whether EVs could render tumors susceptible to immune checkpoint therapy, mice were engrafted with OVA-expressing B16 melanoma cells and, when tumors were palpable, treated with EVs, anti–PD-1/anti–PD-L1, or combination therapy (Fig. 2A). Tumor development was monitored, and mice were sacrificed on day 20 to evaluate immune responses. Monotherapy with anti–PD-1 or anti–PD-L1 was not effective in preventing tumor development because the tumor size averaged 508 ± 356 mm^3^ and 500 ± 208 mm^3^, respectively, whereas mice receiving EV alone or in combination with checkpoint blockade antibodies had tumors with an average size smaller than 100 mm^3^. Tumor sizes in the control group were significantly larger than those in mice that received a combination of EVs and anti–PD-1 or anti–PD-L1 (Fig. 2B).

Splenocytes were analyzed by FACS (gating strategy shown in Supplementary Fig. S2). Splenocytes from mice that received EVs in combination with anti–PD-1 or anti–PD-L1 had significantly higher numbers of antigen-specific CD8^+^ T cells than untreated mice (Fig. 2C). Indeed, mice treated with combination of EVs and anti–PD-1 or anti–PD-L1 had almost twice the number of splenic antigen-specific CD8^+^ T cells (15,152 ± 5,990/spleen and 13,589 ± 2,186/spleen, respectively) versus mice treated with EV alone (7,389 ± 2,591/spleen). Although this difference was not statistically significant, it indicated a tendency of antigen-specific immune activation in response to combination therapy (Fig. 2C). Despite the substantial reduction in tumor growth and increased number OVA-specific T cells upon EV treatment alone, only the combination therapies reached statistical significance compared with PBS (Fig. 2B and C). *Ex vivo* stimulation of splenocytes with the MHC Class I—restricted CD8 OVA peptide and the whole OVA protein induced significantly higher numbers of IFNγ-producing cells in mice that received EVs in combination with either anti–PD-1 or anti–PD-L1 (Fig. 2D). This effect was not observed when splenocytes were stimulated with the MHC Class II—restricted peptide OVA_323–339_ (CD4 peptide), indicating that the combination treatment mainly promoted CD8^+^ T-cell responses.

Next, we sought to determine whether the delay in tumor progression could be due to the recognition of tumor cells by T cells. Thus, splenocytes were restimulated with OVA-expressing B16 cells. Although no significant difference was detected between the groups, a positive trend in IFNγ-producing cells was evident in mice that received EV alone or in combination with checkpoint blockade. In summary, the combination treatment significantly reduced tumor growth and led to significantly more intratumoral antigen-specific T cells than the control group. We conclude that combination therapy has the potential to favor EV-induced antitumor immune responses.

### EV treatment alone or in combination with checkpoint therapy delays tumor development in a melanoma treatment model

**Figure 3.**
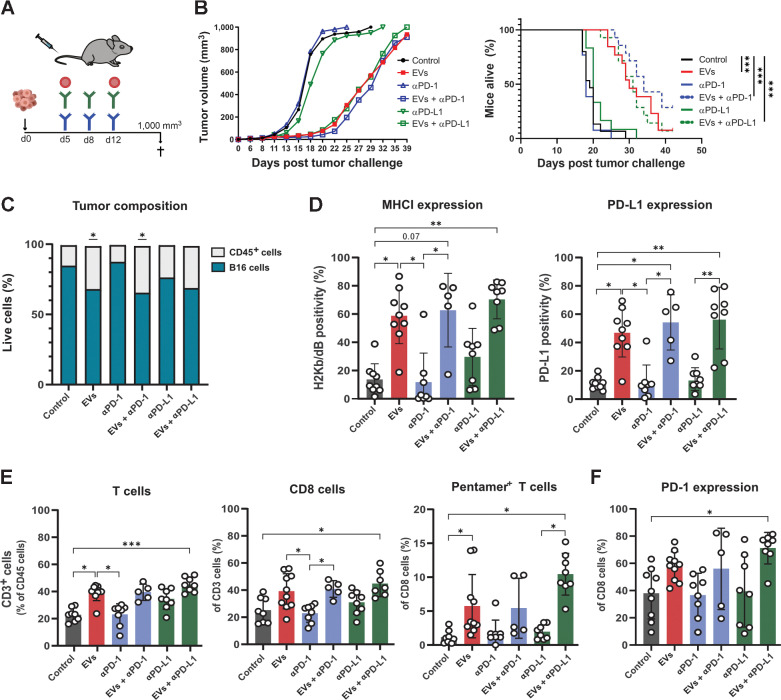
EVs alone or combined with checkpoint blockade treatment induces antigen-specific immune responses in a melanoma model. **A,** Mice with OVA-expressing B16 melanoma were treated with PBS or EVs intravenously and anti–PD-1/PD-L1 intraperitoneally as indicated. **B,** After inoculation, tumors were measured every 2 to 3 days, and mice were sacrificed when the tumors reached 1,000 mm^3^ in size. The results represent the mean size of the tumors in mice in each group (*n* = 10–14). Survival was plotted using a Kaplan–Meier survival curve. **C,** At the endpoint, tumors were collected and analyzed for immune cell infiltration using flow cytometry. Immune cells were identified as CD45^+^ and B16 melanoma cells as CD45^−^. * represents significance compared with the control group. **D,** B16 melanoma cells were analyzed for surface MHC class I and PD-L1 expression. **E,** The proportion of infiltrating total, CD8^+^ and antigen-specific T cells in tumors was analyzed by flow cytometry. **F,** PD-1 expression on the infiltrating CD8^+^ T cells was determined using flow cytometry. The graphs show the results of two independent experiments, with 4 to 5 mice per group. Dots represent a single mouse, and data are presented as the mean ± SD. Data were analyzed using the Kruskal–Wallis test with Dunn test for multiple comparisons. The Mantel–Cox test was applied for the survival curve. *, *P* < 0.05; **, *P* < 0.01; and ***, *P* < 0.001.

On the basis of the data suggesting that an enhanced immune response against B16 melanoma could be generated when EVs were combined with checkpoint blockade, we explored the effect of combination treatment on survival (Fig. 3A). The addition of either anti–PD-1 or anti–PD-L1 to the treatment regimen with EVs did not significantly inhibit tumor growth or prolong survival compared with EV treatment alone (Fig. 3B, left plot). However, four mice were tumor-free after receiving EVs plus anti–PD-1 over 40 days (Fig. 3B, right plot), compared with one mouse in the EV alone and EVs plus anti–PD-L1 treated group. When tumor cells were analyzed by FACS, significantly more tumor-infiltrating immune cells were detected in the EV alone and anti–PD-1 combination groups than in the control group (Fig. 3C). Furthermore, a significantly higher frequency of tumor cells expressing MHC class I and PD-L1 was observed in mice treated with EV alone or in combination with anti–PD-1 and anti–PD-L1 than in other groups (Fig. 3D). When tumor-infiltrating immune cells were analyzed, significantly increased total T-cell and CD8^+^ T-cell infiltration into tumors was detected in response to EV alone or in combination with anti–PD-L1 treatment compared with controls (Fig. 3E). Moreover, the frequency of antigen-specific CD8^+^ T cells was significantly higher in these groups than that in the control group (gating strategy in Supplementary Fig. S3). When we determined PD-1 expression on infiltrating CD8^+^ T cells, a significantly higher expression was observed in the EV and anti–PD-L1 combination groups (Fig. 3F), indicating an enhanced target for anti–PD-1 and anti–PD-L1 treatment.

Because no improvement in survival was observed in the combination groups, despite that more antigen-specific T cells and antigen-responsive cells were observed in these groups (Fig. 2D), we hypothesized that immunosuppressive cells in the tumor microenvironment that could prevent the effects of the combination therapy (gating strategy in Supplementary Fig. S4A). In the combination groups, higher proportions of monocytic cells and lower proportions of granulocytic cells were observed (Supplementary Fig. S4B and S4C). The proportion of macrophages was similar between all groups, indicating that no major differences in the tumor microenvironment (Supplementary Fig. S4D). These data show that the injection of antigen-loaded EVs can induce immune cell infiltration into tumors, specifically CD8^+^ T cells, in addition to increased MHC class I expression on tumor cells in the EV-treated groups, which is a requirement for CD8^+^ T cells to recognize and kill tumor cells.

### Immunization with EVs prior to tumor inoculation sensitizes B16 melanoma tumor cells to anti–PD-1 and anti–PD-L1 therapy

**Figure 4.**
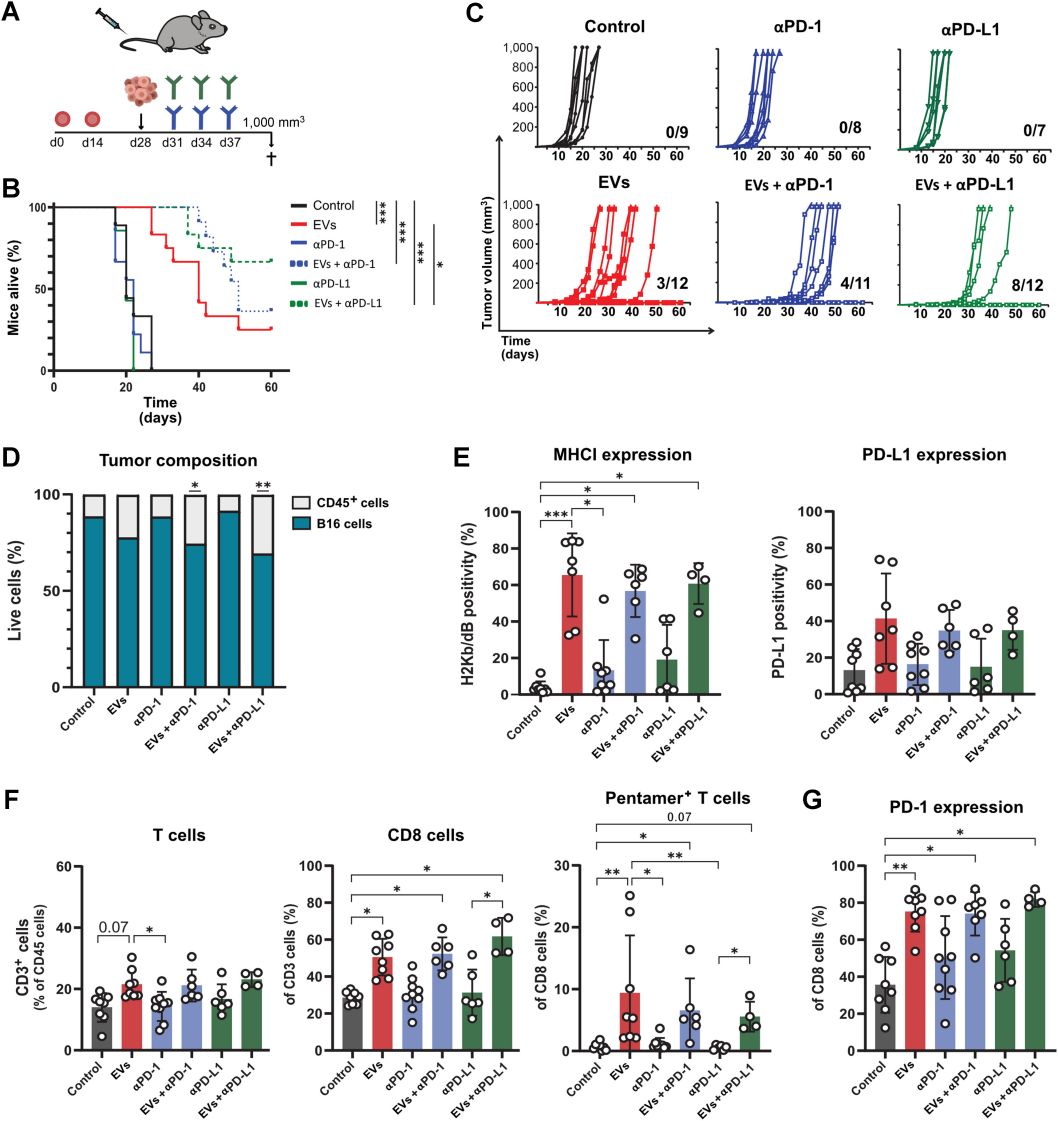
Prophylactic administration of EVs and subsequent injection of checkpoint blockade results in a combination effect and improves survival. **A,** Mice were injected with EVs intravenously before they received OVA-expressing B16 melanoma intraperitoneally, to induce memory T-cell responses as indicated. The mice were sacrificed when tumors reached 1,000 mm^3^. **B,** Survival was plotted using the Kaplan–Meier survival curve. **C,** After inoculation, tumors were measured every 2 to 3 days, and individual tumor growth curves for each group were plotted. Numbers at the bottom right represent surviving mice of the total mice in each group. **D,** At the endpoint, tumors were collected and analyzed for immune cell infiltration using flow cytometry. Immune cells were identified as CD45^+^ cells and B16 melanoma cells as CD45^–^. **E,** B16 melanoma cells were analyzed for surface MHC class I and PD-L1 expression. **F,** The frequency of infiltrating of total, CD8^+^ and antigen-specific T cells in tumors was analyzed by flow cytometry. **G,** PD-1 expression on the infiltrating CD8^+^ T cells was determined using flow cytometry. Graphs show the results of two independent experiments. The experiment included 7 to 12 mice per group, of which 4 to 8 mice per group qualified for flow cytometric analysis of the tumor. Dots represent a single mouse, and data are presented as the mean ± SD. Data were analyzed using the Kruskal–Wallis test with Dunn test for multiple comparisons. The Mantel–Cox test was applied for the survival curve. *, *P* <0.05; **, *P* < 0.01; and ***, *P* < 0.001.

The combination effect on immune responses but not on survival in the therapeutic model could either be due to a low effect of EVs on APCs or because the tumor model was too short for the induced immune response to influence tumor killing. To understand the effect of EVs on APCs and whether they could induce susceptibility of APCs to checkpoint therapy, OVA- and αGC-loaded EVs were added to immature BMDCs which had been cultured for seven days. FACS analysis of the BMDCs after 24 hours showed that OVA- and αGC-loaded EVs were able to induce significant upregulation of PD-L1 and other co-stimulatory molecules such as CD86, CD1d, and ICAM-1 compared with controls (Supplementary Fig. S5). Therefore, we hypothesized that the upregulation of PD-L1 on the BMDCs provided a target for anti–PD-1 or anti–PD-L1 therapy and that checkpoint blockade can further enhance the effect of EVs in a longer tumor model. To test this hypothesis, mice were immunized with antigen-loaded EVs prior to tumor inoculation and then treated with anti–PD-1 or anti–PD-L1 (Fig. 4A). All groups receiving EVs survived longer and showed reduced tumor growth compared with the control group (Fig. 4B; Supplementary Fig. S6A). Mice receiving EVs and the anti–PD-L1 combination showed significantly increased survival compared with the EV group. Furthermore, 3 of 12 mice survived in the group receiving EV alone, whereas 8 of 12 mice survived in the EV and anti–PD-L1 combination group (Fig. 4C). When immune cell infiltration was analyzed, significantly more CD45^+^ cells were detected in tumors from the groups treated with EV and anti–PD-1 or anti–PD-L1 than in controls (Fig. 4D). To determine whether there were more differences between surviving and non-surviving mice, we analyzed the spleens of mice treated with EV alone and EVs in combination with anti–PD-1 or anti–PD-L1 (Supplementary Fig. S6B). When the proportions of T cells were compared between mice with and without tumors, we observed that mice with no tumor growth had significantly more splenic T cells. Next, B16 melanoma cells were analyzed, and all groups that received EVs had tumor cells with significantly higher MHC class I expression. However, compared with previous data, PD-L1 expression was not upregulated in the EV-treated groups (Fig. 4E). When the proportion of T cells was measured, only the groups that received EVs had a significantly higher percentage of T cells in tumors than controls. Nevertheless, the proportions of CD8^+^ T cells and antigen-specific CD8^+^ T cells were significantly higher in the EV and combination groups (Fig. 4F). Finally, PD-1 expression on infiltrating CD8^+^ T cells was measured. All groups that received EVs had significantly higher numbers of PD-1^+^CD8^+^ T cells than the control group (Fig. 4G).

Improved survival in the prophylactic model could be due to EVs inducing T cells to secrete IFNγ, thereby leading to upregulation of MHC class I expression on melanoma cells. Therefore, we stimulated B16 melanoma cells with IFNγ for 24 hours to upregulate MHC class I expression (Supplementary Fig. S7A). Next, mice were inoculated with the IFNγ-stimulated or unstimulated B16 melanoma cells, followed by anti–PD-1 or anti–PD-L1 treatment (Supplementary Fig. S7B). No prolonged survival was observed in response to checkpoint blockade in mice that received IFNγ-stimulated B16 melanoma cells compared with the mice inoculated with unstimulated B16 melanoma cells, suggesting that the improved survival of the EV-treated groups was not due to the presence of MHC class I on the melanoma cells at the time of anti–PD-1/anti–PD-L1 injection (Supplementary Fig. S7C and S7D). Another reason for the lack of tumor growth in the prophylactic, but not therapeutic model, could be the different timepoints of EV and antibody injections. However, when mice were first treated with EVs before anti–PD-1/anti–PD-L1 treatment in the therapeutic model, no delay in tumor growth or improvement in mouse survival was observed (Supplementary Fig. S8A-S8C). These data support that the effect observed in the prophylactic model was not due to the timing of EVs and anti–PD-1/anti–PD-L1 or the pre-presence of MHC class I on the tumor cells. Overall, our findings indicate that antigen-loaded EVs can induce potent immune responses that can turn nonresponsive/refractory tumor cells into anti–PD-1/anti–PD-L1 treatment–sensitive cells.

## Discussion

During the last decade, checkpoint blockade has significantly improved cancer treatment. Although a subset of patients shows extraordinary responses to anti–PD-1 or anti–PD-L1 treatment, the majority of patients only benefit partly, temporarily, or do not respond at all (28). For checkpoint blockade to work in nonresponsive patients, a combination therapy that can promote immunogenicity of the tumor or induce more robust antitumor immunity is needed. In this study, we showed that antigen-loaded EVs could induce tumor-specific T cells, promote immune infiltration into tumors, and induce MHC class I expression on the tumor cells. Finally, in a prophylactic tumor model, EVs could turn a tumor that was nonresponsive to anti–PD-L1 into a responsive tumor, thereby prolonging survival. Because increased immune responses after combination therapy were also observed in the treatment models, we propose that the lack of a combination effect on survival in the treatment models was due to the longer timespan of the prophylactic model than its prophylactic nature, making it relevant for human immunotherapy.

To mimic patients who are nonresponsive to anti–PD-1 or anti–PD-L1 treatment, we used a B16 melanoma model that was not responsive to checkpoint treatment alone. Although research has shown that B16 melanoma can respond to anti–PD-1 and anti–PD-L1 treatment alone (29, 30), several studies have shown little or no response in this tumor model (23, 24, 31, 32). These differences in responsiveness might be explained by the fact that different clones have been used in different studies. Furthermore, the microbiota, which can vary among different animal facilities, has also been shown to affect the response to anti–PD-L1 (33).

One of the reasons why B16 melanoma does not respond to checkpoint blockade is that unlike other tumor models such as MC38, it expresses low MHC class I (34, 35). Indeed, MHC class I expression by tumor cells seems to be an important criterion for the effectiveness of checkpoint blockade (36, 37). However, treatment with IFNγ can induce MHC class I expression on B16 melanoma cells, as shown here and by others (38). In the current study, we showed that antigen-loaded EVs induced MHC class I expression on B16 melanoma cells *in vivo*. The mechanism by which EVs do this remains to be examined, but it is likely to be via the induction of IFNγ. Previously, we showed that antigen-loaded EVs can activate NK and iNKT cells, which are known producers of IFNγ. Furthermore, our data showed that antigen-loaded EV treatment implicitly induced T-cell activation and infiltration into tumors, where these cells can secrete IFNγ. However, pretreatment of the tumor cells with IFNγ did not lead to increased responsiveness to checkpoint treatment; thus, the effect was not fully explained by increased MHC class I on the tumor cells, but rather activation of a broad range of immune cells.

An additional reason why B16 melanoma does not respond to checkpoint blockade might be that the immune cell infiltrate in this model is low compared with other tumor models such as RENCA, 4T1, and CT26 (39, 40). It has previously been shown that therapeutic anti–PD-1 responses associate with the presence of CD8^+^ T cells at the tumor margin before therapy, colocalizing with PD-L1–expressing tumors (41). Here, we showed that treatment with antigen-loaded EVs induced significantly higher immune cell infiltration into tumors compared with controls. Furthermore, tumors of mice treated with EVs had significantly higher proportions of CD8^+^ T cells and antigen-specific T cells, indicating that EVs not only promoted immune cell infiltration, but also infiltration of tumor-specific T cells. In addition, infiltrating CD8^+^ T cells in the groups that received EV treatment showed a higher expression of PD-1. Although PD-1 expression on T cells was initially seen as a marker of exhaustion, it is becoming clear that PD-1 is a marker of activation and is mainly expressed by tumor-reactive T cells (42–44).

Although antigen-loaded EVs promoted the immunogenicity of B16 melanoma and immune cell infiltration in this model, we did not observe an improved response to EVs and anti–PD-1/anti–PD-L1 combination treatment on survival in the treatment model compared with EVs alone. An explanation for this might be that this tumor model is aggressive. In regards to tumor growth, a rapid effect of EV treatment and reduced tumor growth were observed as soon as 5 days after the first EV injection. This indicates that EVs also activate the innate immune system, as fully functional adaptive immune responses can take up to 2 weeks to develop. It is plausible that this model is too short for the CD8^+^ T-cell response to have a detectable effect. Consequently, the administration of anti–PD-1 and anti–PD-L1 will not be effective, and therefore, cannot reach its full potential. In two published models where anti–PD-L1 or anti–PD-1 had some effect alone, EVs were shown to synergize with checkpoint blockade in a therapeutic model, indicating that an immunogenic tumor will also respond more easily to combination therapy (45, 46).

One limitation of the current study is that the role of EVs released from tumors was not investigated. Previously, it has been shown that tumor-derived EVs carry PD-L1 and can suppress CD8^+^ T cells, and high circulation in plasma correlates with unresponsiveness to anti–PD-1 treatment (47). Furthermore, it has been shown that T cells need to be primed by tumor-derived antigens in the absence of tumor-derived exosomal PD-L1 to become functional T cells (48). In our study, it is possible that naïve T cells had already encountered tumor-derived EVs expressing PD-L1 in a therapeutic model before the treatment started, resulting in suppressed T cells and unresponsiveness to the checkpoint therapy. In the prophylactic tumor model, however, T cells were activated in the absence of a suppressive tumor microenvironment before the tumor was given and its suppressive EVs were present. This could be a possible mechanism by which antigen-loaded EVs induce responsiveness to anti–PD-1/anti–PD-L1 treatment in a refractory tumor model. What could be regarded as another limitation of this study is that unloaded EVs were not included as a control. However, previous publications from our group did compare unloaded EVs, BSA-loaded EVs, or EVs loaded with OVA in peptide form in various *in vitro* and *in vivo* experiments (8, 25). We found that EVs loaded with OVA were superior in inducing OVA-specific T-cell responses, as well as in reducing the growth of B16 tumors in mice. Thus, we considered it very unlikely that unloaded EVs induced an antitumor immune response that could sensitize tumors to checkpoint blockade therapy, and therefore we omitted these groups in the current study.

On the basis of our previous research, EVs can induce antigen-specific immune responses. To allow time for this, we injected antigen-loaded EVs prior to tumor inoculation. This should generate an antitumor immune memory response, which could subsequently contain and suppress tumor growth in early stages, thereby prolonging the tumor model. Using this model, we observed that B16 melanoma cells responded to EV and anti–PD-L1 treatment, as evidenced by reduced tumor growth and significantly prolonged survival compared with the group that received EVs only. Functional anti–PD-L1 might be a direct result of the presence of memory tumor-specific T cells. However, it has recently been shown that preexisting tumor-specific T cells may have limited recovery capacity and that T-cell responses to checkpoint blockade come from different T-cell clones that may have just recently entered the tumor (49). This could mean that the memory T cells that are generated by preventive EV injection are not T-cell clones that respond to checkpoint blockade, but this remains to be elucidated.

Recently, it has been shown that PD-L1 expression on immune cells is critical for the inhibition of antitumor immunity in B16 melanoma, whereas for the MC38 model, only PD-L1 expression on the tumor itself is relevant for immune evasion (50). Our data showed that DCs upregulated PD-L1 upon stimulation with antigen-loaded EVs. Although PD-L1 upregulation might suppress antigen-specific immune responses, the administration of anti–PD-L1 could overcome this effect and thereby promote antitumor immunity in the B16 melanoma model.

In conclusion, this study indicates that antigen-loaded EVs may be effective in settings where checkpoint blockade therapy is unsuccessful. EVs alone were effective in a checkpoint-refractory model. This suggests that EVs provide complementary treatment for checkpoint therapy. The variability of individual tumors and tumor heterogeneity within a tumor support the use of therapies or combinations thereof with diverse effects on the immune system and tumor cells. We observed that EVs induced immune responses that could be boosted by checkpoint blockade therapy, even in a model refractory to checkpoint therapy alone. These data indicate that EV treatment has the potential to synergize with checkpoint blockade therapy in a clinical setting or could be a treatment option for tumors that are unresponsive to checkpoint blockade in humans.

## Supplementary Material

Supplementary Figure Legends Supplementary figure legends

Supplementary Figure 1 BMDC cell surface markers, and characterization of EVs from these cells, additional to Figure 1.

Supplementary Figure 2 Gating strategy for OVA-specific CD8 T cells in splenocytes

Supplementary Figure 3 Gating strategy for MHCI+ and PD-L1+ tumor cells and intratumoral PD-1+ and pentamer+ T cells

Supplementary Figure 4 Gating strategy for monocytes, granulocates and macrophages

Supplementary Figure 5 Upregulation of functional markers on DCs after in vitro exposure to EVs.

Supplementary Figure 6 Mean growth curve of tumors in prophylactic setting and percentage of T cells in splenocytes of mice with vs without tumor development

Supplementary Figure 7 and 8 Figure S7: MHCI and PD-L1 on IFNy-stimulated B16 cells, and tumor growth of these cells. Figure S8: Tumor growth and survival mice after late treatment with anti-PD-1/PD-L1 in combination with EVs
