# Supplementary material for: Antigen-Loaded Extracellular Vesicles Induce Responsiveness to Anti–PD-1 and Anti–PD-L1 Treatment in a Checkpoint Refractory Melanoma Model
Source: Cancer Immunol Res. 2023 Jan 10;11(2):217–27. doi: 10.1158/2326-6066.CIR-22-0540 (PMC9896027; doi:10.1158/2326-6066.CIR-22-0540)
Supplement: Supplementary Figure Legends [file cir-22-0540_supplementary_figure_legends_suppsm1.docx]

**Supplementary Figure Legends**

**Supplementary Figure 1. (**A**)** BMDCs from mice were analyzed for surface markers on day 9 by flow cytometry. Data are presented as MFI ratios between the specific antibody and its corresponding isotype control. Data are presented as mean ±SD, n=8. (B) Transmission electron microscopy pictures of EVs, showing two different magnifications. Red arrows indicate examples of particles with characteristic EV morphology. (C) Proteins isolates from two batches of BMDC cells and EVs were subjected to western blot and stained for calnexin and actin.

**Supplementary Figure 2.** Representative plots show gating strategy for OVA-specific CD8^+^ T cells as B220^-^ CD3^+^ CD8^+^ pentamer^+^ in splenocytes.

**Supplementary Figure 3.** Representative plots show gating strategy for (A) MHCI^+^ and (B) PD-L1^+^ tumor cells represented as CD45^-^ cells; (C) PD-1^+^ and (D) pentamer^+^ CD8^+^ T cells represented as CD45^+^ CD3^+^ NK1.1^-^ CD8^+^ cells.

**Supplementary Figure 4.** (A) Representative plots show gating strategy for monocytes as CD45^+^ CD11b^+^ Ly6C^+^ Ly6G^-^, granulocytes CD45^+^ CD11b^+^ Ly6C^-^ Ly6G^+^ and macrophages CD45^+^ CD11b^+^ Ly6C^-^ Ly6G^+^ F4/80^+^. Bar plots show percentages of (B) monocytic cells, (C) granulocytic cells and (C) macrophages within CD11b^+^ cells in the tumor.

**Supplementary Figure 5. OVA- and αGC-loaded EVs induce upregulation of immune molecules on BMDC.** Bone marrow cells were differentiated into immature BMDCs and at day seven stimulated for 24 h with 1 or 5 µg EVs or positive or negative controls, as indicated. Surface markers were analyzed using flow cytometry. Data present the results of three independent experiments. The dots represent a single EV batch or control, which is the average of duplicate analyses. Data are presented as mean ± SD and were analyzed using Brown-Forsythe and Welch ANOVA test with Dunnett’s test for multiple comparisons. *p <0.05, **p<0.01 and ***p<0.001.

**Supplementary Figure 6.** (A) The graph represents tumor growth in mice after tumor inoculation. Tumors were measured every 2-3 days and mice were sacrificed when the tumors reached 1000 mm^3^ in size. The results represent the mean size of the tumors in mice in each group. (B) Bar plots show T cell percentages in splenocytes from mice with and without tumors. Graphs show the results of two independent experiments. Dots represent a single mouse, and data are presented as the mean ± SD. Data were analyzed using the Mann-Whitney test. *p <0.05, **p<0.01 and ***p<0.001.

**Supplementary Figure 7.** (A) Representative plots show staining against isotype, MHCI^+^ and PD-L1^+^ on OVA expressing B16 cells after IFNγ stimulation. (B) Graph represents tumor growth in mice after tumor inoculation. Tumors were measured every 2-3 days and mice were sacrificed when the tumor reached 1000 mm^3^ in size. Results represent the mean size of tumors in mice in each group. (C) Survival was plotted using a Kaplan–Meier survival curve. n=10.

**Supplementary Figure 8:** (A) Mice with OVA expressing B16 melanoma tumors were treated *i.v*. with EVs followed by *i.p.* αPD-1/ αPD-L1 treatment as indicated. (B) Graph represents tumor growth in mice after tumor inoculation. Tumors were measured every 2-3 days and mice were sacrificed when the tumor reached 1000 mm^3^ in size. Results represent the mean size of tumors in mice in each group. (C) Survival was plotted using a Kaplan–Meier survival curve. n=4-5.
