# Supplementary figures and images for: Antigen-Loaded Extracellular Vesicles Induce Responsiveness to Anti–PD-1 and Anti–PD-L1 Treatment in a Checkpoint Refractory Melanoma Model
Source: Cancer Immunol Res. 2023 Jan 10;11(2):217–27. doi: 10.1158/2326-6066.CIR-22-0540 (PMC9896027; doi:10.1158/2326-6066.CIR-22-0540)

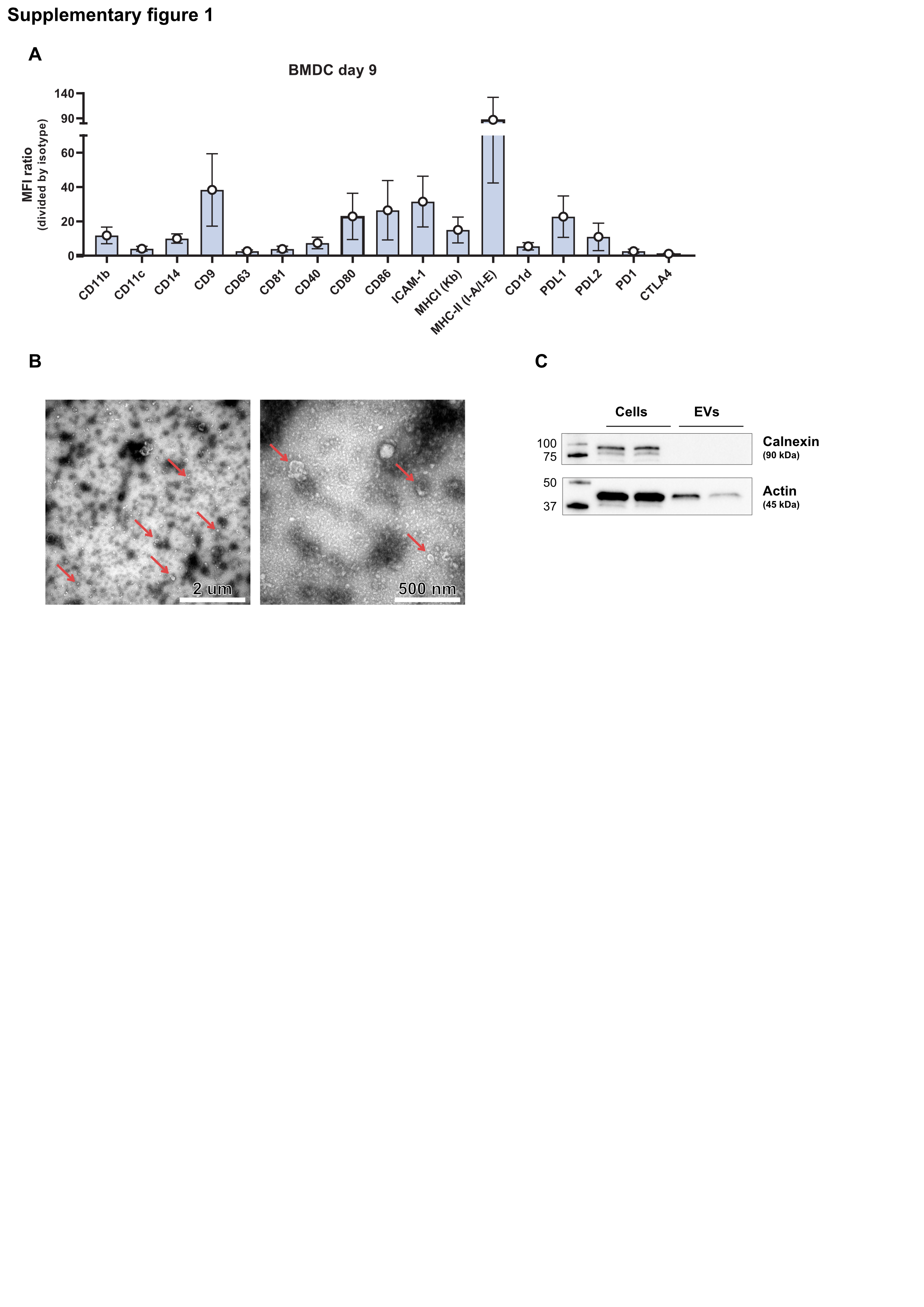

Supplement: Supplementary Figure 1 — BMDC cell surface markers, and characterization of EVs from these cells, additional to Figure 1. [file cir-22-0540_supplementary_figure_1_suppsf1.png]

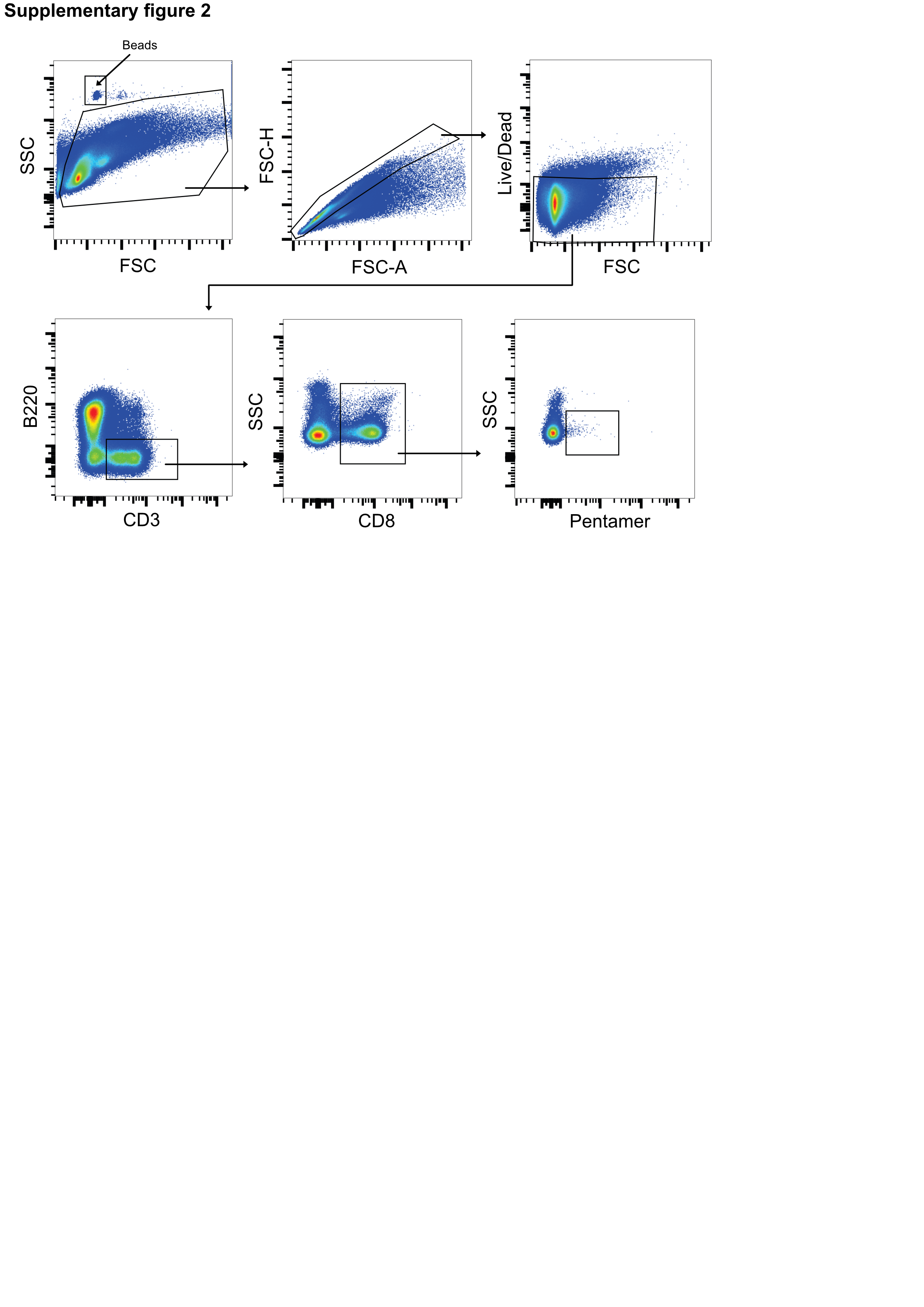

Supplement: Supplementary Figure 2 — Gating strategy for OVA-specific CD8 T cells in splenocytes [file cir-22-0540_supplementary_figure_2_suppsf2.png]

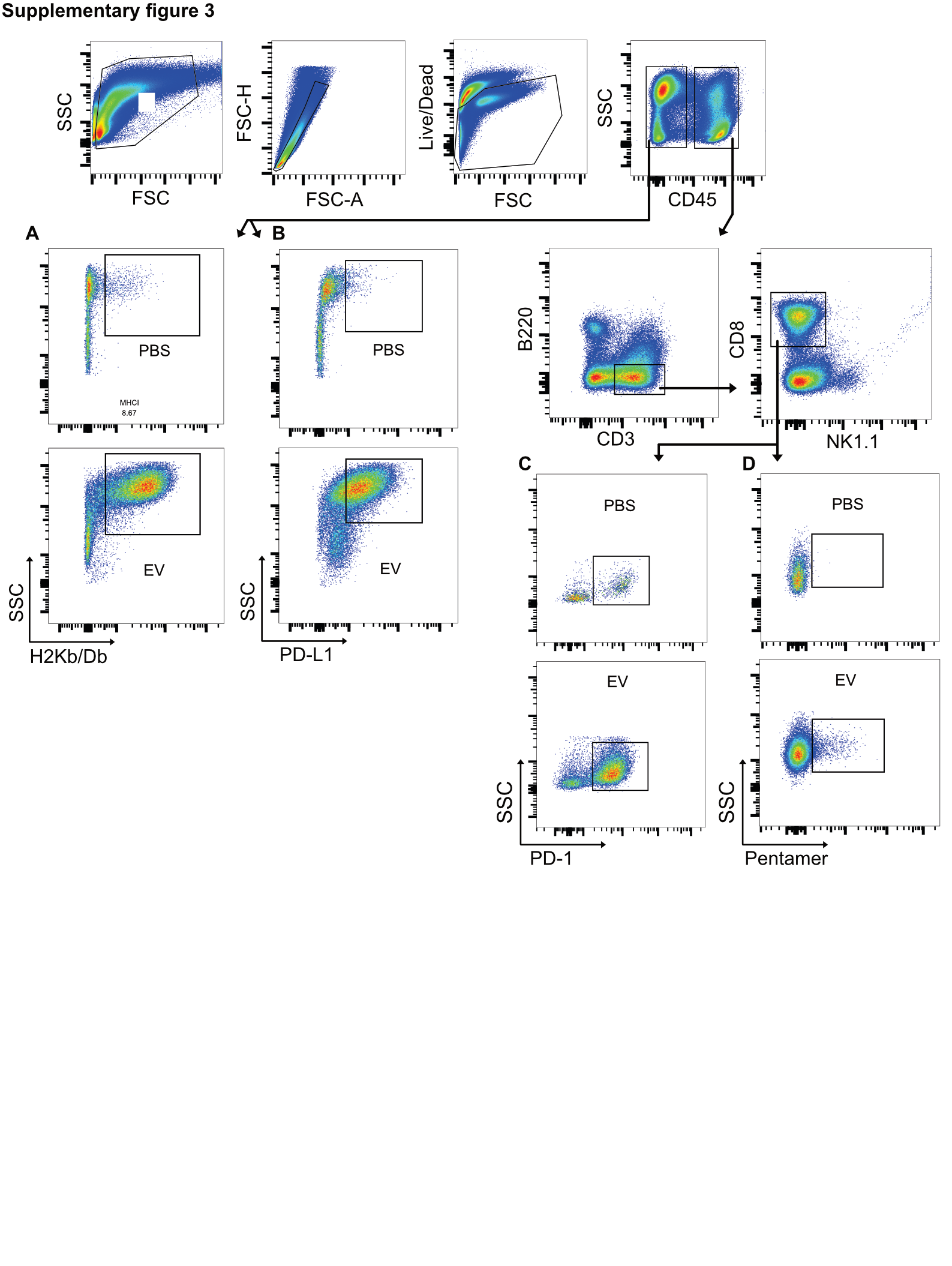

Supplement: Supplementary Figure 3 — Gating strategy for MHCI+ and PD-L1+ tumor cells and intratumoral PD-1+ and pentamer+ T cells [file cir-22-0540_supplementary_figure_3_suppsf3.png]

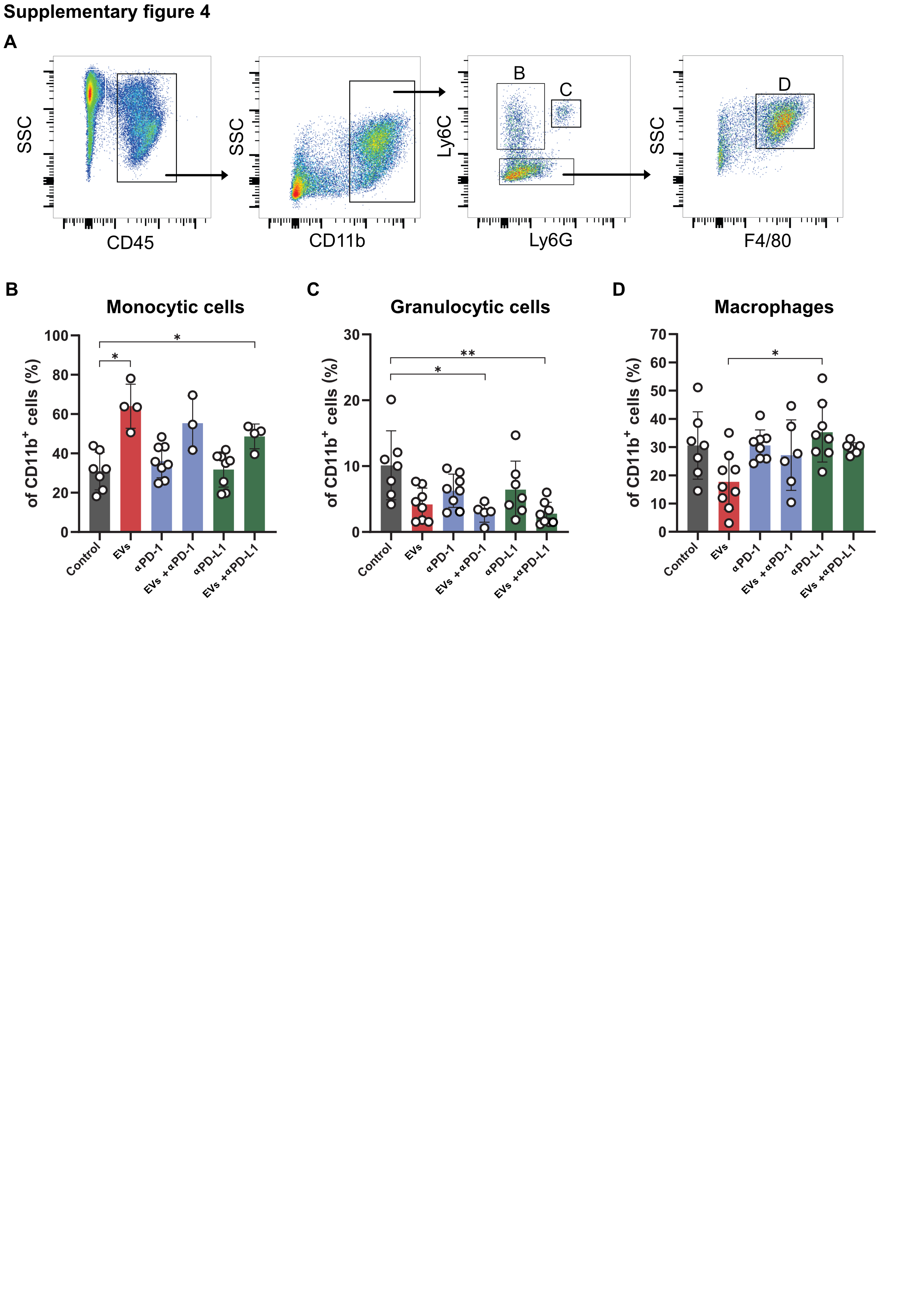

Supplement: Supplementary Figure 4 — Gating strategy for monocytes, granulocates and macrophages [file cir-22-0540_supplementary_figure_4_suppsf4.png]

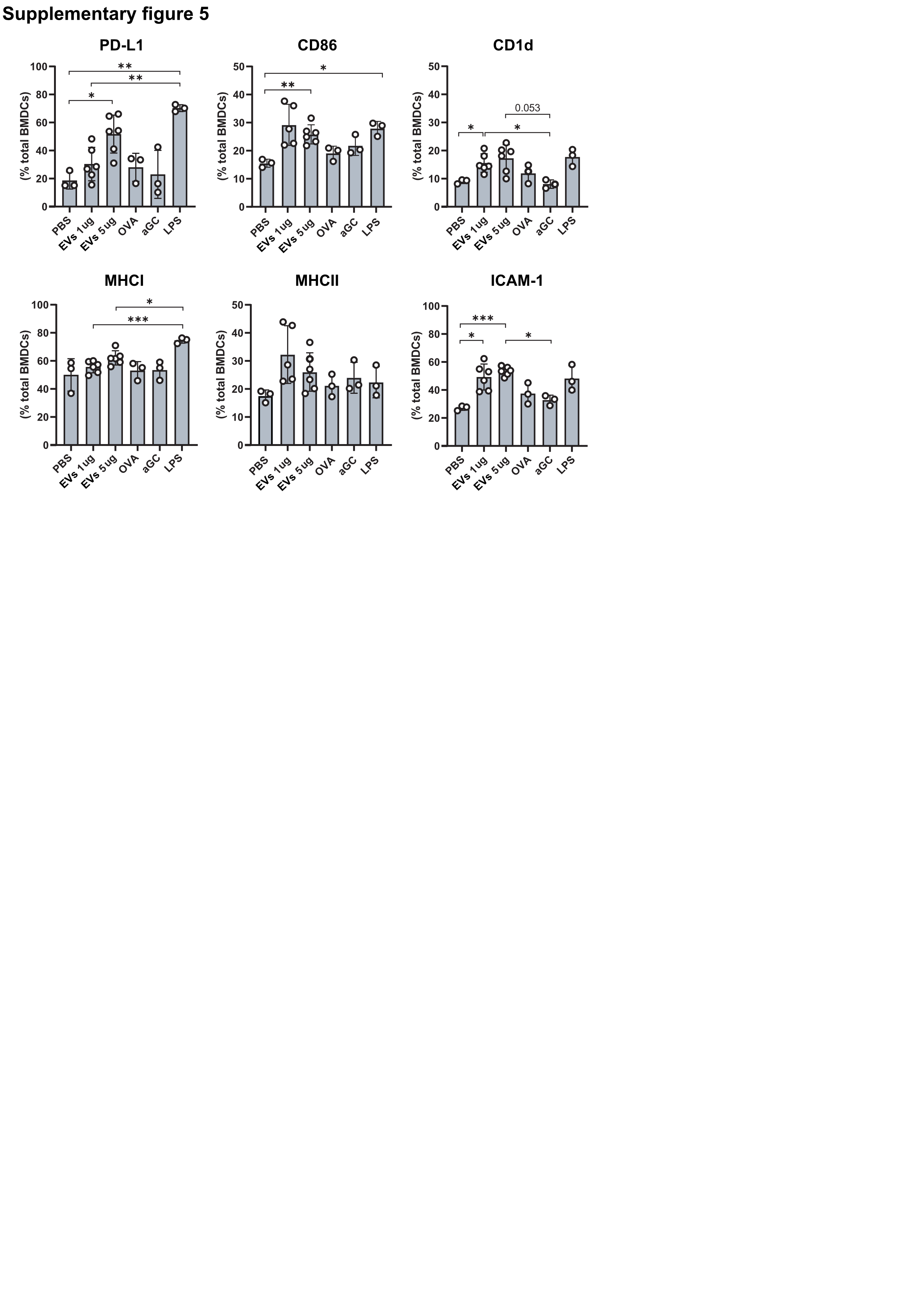

Supplement: Supplementary Figure 5 — Upregulation of functional markers on DCs after in vitro exposure to EVs. [file cir-22-0540_supplementary_figure_5_suppsf5.png]

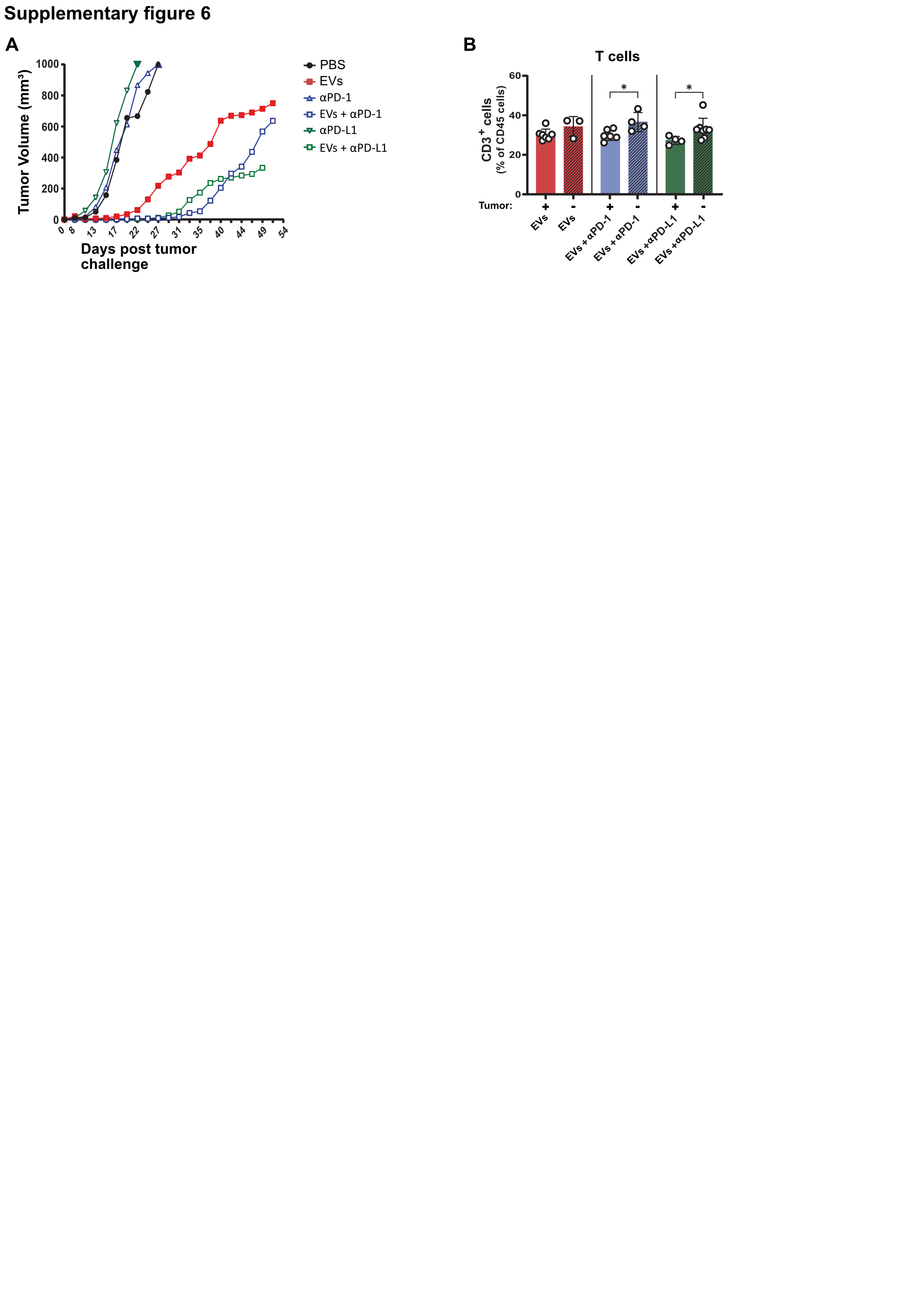

Supplement: Supplementary Figure 6 — Mean growth curve of tumors in prophylactic setting and percentage of T cells in splenocytes of mice with vs without tumor development [file cir-22-0540_supplementary_figure_6_suppsf6.png]

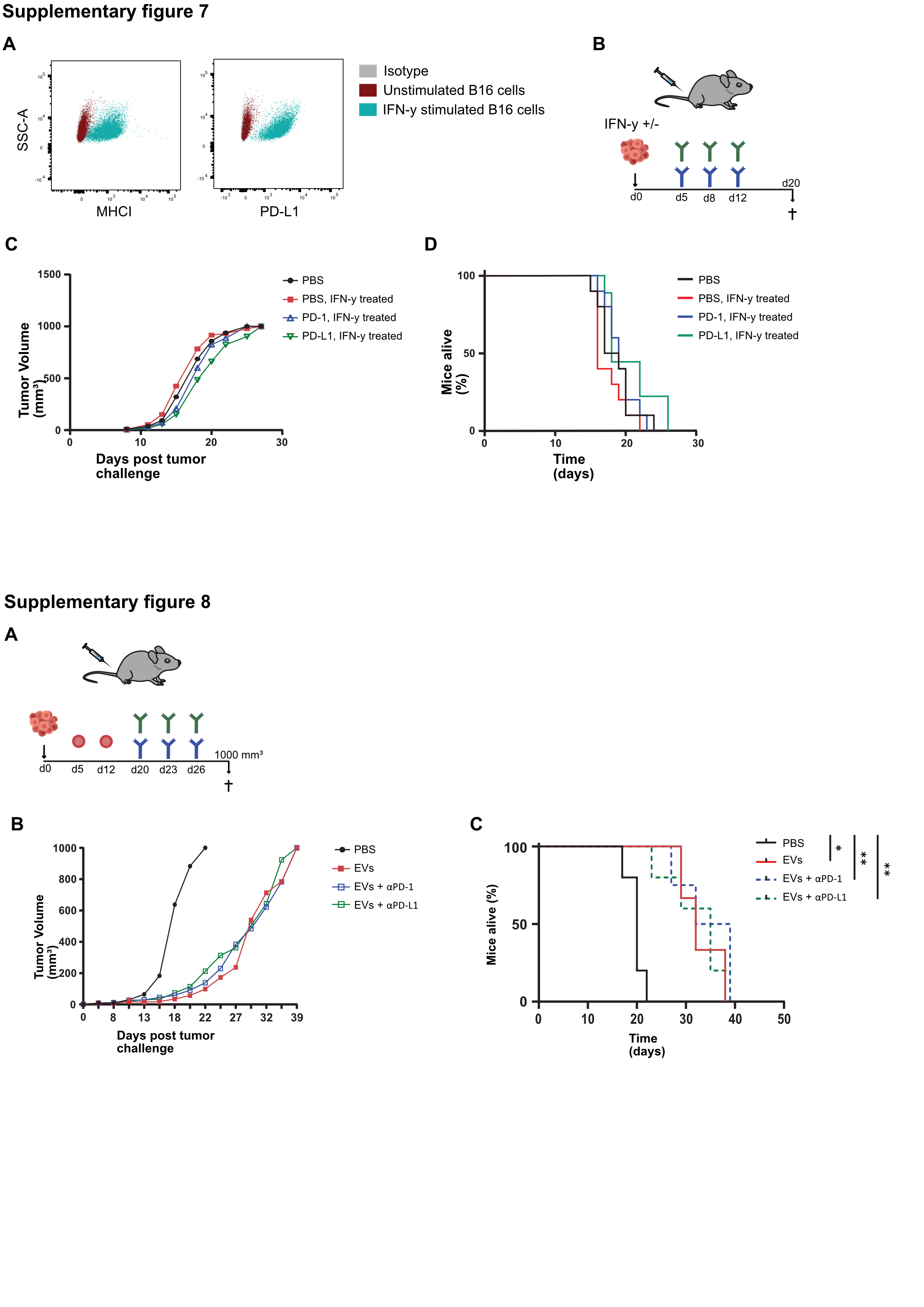

Supplement: Supplementary Figure 7 and 8 — Figure S7: MHCI and PD-L1 on IFNy-stimulated B16 cells, and tumor growth of these cells. Figure S8: Tumor growth and survival mice after late treatment with anti-PD-1/PD-L1 in combination with EVs [file cir-22-0540_supplementary_figure_7_and_8_suppsf7-sf8.png]
